# Distinct Changes in Functional Connectivity in Posteromedial Cortex Subregions during the Progress of Alzheimer’s Disease

**DOI:** 10.3389/fnana.2016.00041

**Published:** 2016-04-12

**Authors:** Yan Wu, Yaqin Zhang, Yong Liu, Jieqiong Liu, Yunyun Duan, Xuehu Wei, Junjie Zhuo, Kuncheng Li, Xinqin Zhang, Chunshui Yu, Jiaojian Wang, Tianzi Jiang

**Affiliations:** ^1^Key Laboratory for NeuroInformation of Ministry of Education, School of Life Science and Technology, University of Electronic Science and Technology of ChinaChengdu, China; ^2^Brainnetome Center, Chinese Academy of SciencesBeijing, China; ^3^National Laboratory of Pattern Recognition, Institute of Automation, Chinese Academy of SciencesBeijing, China; ^4^Department of Neurology, Xuanwu Hospital of Capital Medical UniversityBeijing, China; ^5^Department of Radiology, Xuanwu Hospital of Capital Medical UniversityBeijing, China; ^6^Department of Radiology, Tianjin Medical University General HospitalTianjin, China; ^7^The Queensland Brain Institute, The University of QueenslandBrisbane, QLD, Australia; ^8^CAS Center for Excellence in Brain Science, Institute of Automation, Chinese Academy of SciencesBeijing, China

**Keywords:** Alzheimer’s disease, posteromedial cortex, functional connectivity, network, subregions

## Abstract

Alzheimer’s disease (AD) is a progressive neurodegenerative disorder which causes dementia, especially in the elderly. The posteromedial cortex (PMC), which consists of several subregions involved in distinct functions, is one of the critical regions associated with the progression and severity of AD. However, previous studies always ignored the heterogeneity of the PMC and focused on one stage of AD. Using resting-state functional magnetic resonance imaging, we studied the respective alterations of each subregion within the PMC along the progression of AD. Our data set consisted of 21 healthy controls, 18 patients with mild cognitive impairment (MCI), 17 patients with mild AD (mAD), and 18 patients with severe AD (sAD). We investigated the functional alterations of each subregion within the PMC in different stages of AD. We found that subregions within the PMC have differential vulnerability in AD. Disruptions in functional connectivity began in the transition area between the precuneus and the posterior cingulate cortex (PCC) and then extended to other subregions of the PMC. In addition, each of these subregions was associated with distinct alterations in the functional networks that we were able to relate to AD. Our research demonstrated functional changes within the PMC in the progression of AD and may elucidate potential biomarkers for clinical applications.

## Introduction

Alzheimer’s disease (AD) is a neurodegenerative disorder clinically characterized by progressive cognitive decline and neuropsychiatric symptoms (Ballard et al., 2011). Memory loss is the predominant symptom in mild cognitive impairment (MCI), which is seen as the prodromal stage of AD (Petersen et al., 1999; Petersen, 2009). Patients with mild AD (mAD) can be impaired in their language, executive, visuospatial, and perceptive functions, as well as in other cognitive and behavioral domains (Dubois et al., 2007, 2010; Sheline and Raichle, 2013). Some patients become irritable, aggressive, have mood swings, and/or wander away from family and society (Giaccone et al., 2011). As the disease progresses, patients can lose their bodily functions and become completely dependent upon caregivers (Dubois et al., 2010).

The posteromedial cortex (PMC) is one of the critical regions associated with the progression and severity of AD. In subjects at risk for AD, the PMC demonstrates reduced task-induced deactivation in apolipoprotein E(APOE) ε4 allele carriers compared to noncarriers (Pihlajamäki et al., 2010). In the early stages of AD, the PMC shows the earliest and largest decrement in energy metabolism, even with no clinical symptoms (Valla et al., 2001). In the later stages of AD, connections between the PMC and other functional networks, such as the memory and default mode network (DMN), and high vision-related functions and olfaction (Zhang et al., 2009; Wu et al., 2011), are disrupted. However, how the time course of the dynamic alterations in the PMC functional connectivity correspond with AD progression remains unknown. Previous studies have verified that the PMC is a heterogeneous structure consisting of several subregions. The dorsal-anterior, dorsal-central, and dorsal-posterior parts of the PMC participate in sensorimotor, cognitive/associative, and visual functions, respectively. The ventral PMC contains a transitional region and a core part of the DMN (Margulies et al., 2009; Zhang et al., 2014). Given their distinct connection profiles and functions, the subregions of the PMC may differ in their vulnerability to AD and may play different roles in AD processing changes. Studying the respective alterations of each PMC subregion throughout the evolution of AD will provide further insight into the mechanisms of the functional changes in the PMC that occur with AD progression and may lead to identifying potential biomarkers that could be used in clinical situations.

Using the new PMC atlas (Zhang et al., 2014), the present study focused on the distinct functional abnormalities in each of the PMC subregions with the progression of AD. Since MCI is a transitional state between normal aging and dementia is a high risk factor for developing AD, an MCI group was included in our research. Therefore, alterations in functional connectivity were investigated in each PMC subregion in MCI, mAD, and severe AD (sAD) groups as well as in a healthy control group.

## Materials and Methods

### Subjects

All the participants in the present study have been described in detail in our previous research (Song et al., 2013; He et al., 2014; Liu et al., 2014). The participants were recruited by advertisement, and they and various aspects of the study were supported by medical personnel from a neuropsychological research facility at Xuanwu Hospital, Beijing, China. None of the participants had any contraindications for MRI scanning. Written consent forms were obtained from all subjects or their legal guardians (usually a family member). The study was approved by the ethics committee of Xuanwu Hospital. Following the criteria of our previous studies, 18 patients with MCI, 17 patients with mAD, 18 patients with sAD, and 21 healthy controls were all included in the present study. The AD patients were diagnosed using standard operationalized criteria DSM-IV (Diagnostic and Statistical Manual of Mental Disorders, Fourth Edition); NINCDS-ADRDA (American Psychiatric Association, 1994 and National Institute of Neurological and Communicative Disorders and Stroke—AD and Related Disorders Association); (McKhann et al., 1984). The severity of dementia was assessed using the clinical dementia rating (CDR) scale (Morris, 1993). AD patients with a CDR score of 1 were diagnosed as having mAD and those with a CDR score of 2 or 3 were diagnosed as having sAD. MCI was diagnosed using standard criteria (Petersen et al., 1999, 2001; Choo et al., 2007), which included subjective memory loss with objective evidence of memory impairment in the context of normal or near-normal performance on other domains of cognitive functioning; minimal impairment of activities of daily living; and a CDR score of 0.5. The healthy controls had a CDR score of zero. None of the subjects had head movements of >3 mm translation or >3° angular rotation in any axis during scanning. Demographic and psychological characteristics of the samples are summarized in Table 1.

**Table 1:** **Demographics and clinical characteristics**.

	HC (*n* = 21)	MCI (*n* = 18)	mAD (*n* = 17)	sAD (*n* = 18)	*p*-value	
Gender (M/F)	7/14	10/8	8/9	9/9	0.543
Age (years)	65.0 ± 8.1	70.2 ± 7.9	66.1 ± 8.3	65.4 ± 8.6	0.219
Education (years)	11.0 ± 4.4	9.4 ± 4.8	10.4 ± 4.2	10.9 ± 4.3	0.690
MMSE	28.5 ± 1.4	21.9 ± 5.0	14.3 ± 5.8	6.2 ± 4.9	<0.001
CDR	0	0.5	1.0	2.2 ± 0.4	–

*Note: A Pearson chi-squared test was used for gender comparison. One-way ANOVAs with Bonferroni-corrected post hoc t-tests were used for age, education and MMSE comparisons. MMSE, mini-mental state examination; CDR, clinical dementia rating*.

### Data Acquisition

The functional MR images were acquired on a 3.0 Tesla MR scanner (Magnetom Trio, Siemens, Germany) with an echo planar imaging sequence sensitive to blood oxygenation level dependent (BOLD) contrast: repetition time (TR) = 2000 ms, echo time (TE) = 30 ms, flip angle = 90°, matrix = 64 × 64, field of view (FOV) = 220 mm ×220 mm, slice thickness = 3 mm with inter-slice gap = 1 mm. Each brain volume comprised 32 axial slices and each scanning session lasted for 360 s. Sagittal 3D T1-weighted images were also acquired (TR/TE = 2000/2.6 ms; flip angle = 9°; FOV = 256 mm × 224 mm; matrix = 256 × 224; slice thickness = 1 mm, no gap; 176 sagittal slices).

### VBM Analyses

Voxel-based morphometric analyses were performed to determine the structural changes between MCI, mAD, sAD and normal controls. The structural MRI images were preprocessed using VBM8 toolbox^1^ in SPM8 (Wellcome Department of Imaging Neuroscience Group, UK^2^). The modulated VBM8 were used to calculate the gray matter volume and was corrected for total intracranial volume. Each structural image was transformed to Montreal Neurological Institute (MNI) space using DARTEL-normalized and subsequently was segmented into gray matter, white matter and cerebrospinal fluid using a fully automated algorithm within SPM8. Next, the normalized gray matter images were smoothed with an 8 mm full-width at half-maximum (FWHM) Gaussian kernel for the subsequent statistical analyses (Ashburner and Friston, 2000). A two sample *t*-test (with age, gender and educational year treated as covariates) was performed on these normalized gray matter volume maps to determine areas with significant differences in gray matter volume between each of the patient groups and the healthy controls. Finally, voxel-wise family wise error (FWE) method was used for multi-comparison correction to control type 1 error (*p* < 0.05, FWE corrected).

### Data Preprocessing

Preprocessing of the functional data was carried out by AFNI^3^ and FSL^4^. The first 10 images were discarded to allow the signal to reach equilibrium. The remaining 170 images were first corrected for within-scan acquisition time (TA) differences between slices and then realigned to the first volume to correct for intrascan head motion. Motion time courses were obtained by estimating the values for translation and rotation for each of the 170 consecutive volumes. Next, the functional images were spatially smoothed with a Gaussian kernel of 6 × 6 × 6 mm^3^ to decrease spatial noise. The functional MRI waveform of each voxel was temporally band-pass filtered (0.01 Hz < *f* < 0.08 Hz) and the linear drift of the signal was removed. The preprocessing steps described above were performed using AFNI. Subsequently, we spatially registered the realigned, smoothed and temporally filtered images to the MNI space and resampled them to 2 × 2 × 2 mm^3^ by FSL. Several sources of spurious or regionally nonspecific variance, including six parameters indicative of rigid body/head motion (obtained from motion correction), the signal averaged over the whole-brain, the signal averaged over the lateral ventricles, and the signal averaged over a region centered in the deep cerebral white matter, were then removed by regression as nuisance variables. In addition, considering the whole-brain signal regression will exaggerate anti-correlation, we also repeat the analyses using the fMRI data without global signal regression to ensure that the obtained results are reliable.

### Functional Connectivity and Statistical Analysis

**Figure 1:**
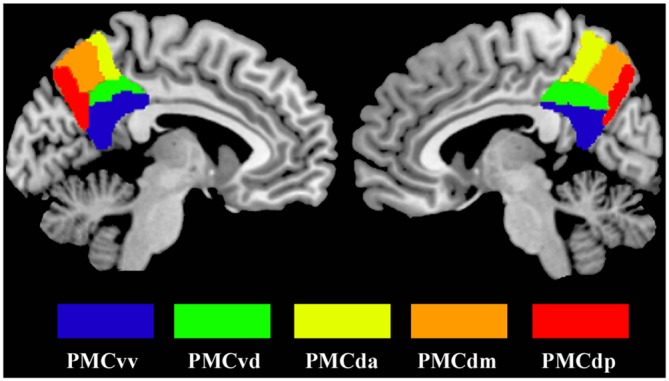
**The maximum probability maps of the subregions defined within the human posteromedial cortex (PMC), displayed on a medial surface view using the colin27 template.** The maximum probability maps for each subregion of PMC were obtained using anatomical connectivity patterns based parcellation in our previous study.

Seed regions in the PMC were defined by the 50% probability map in the PMC atlas from a previous study (Zhang et al., 2014). Five subregions (that’s PMCda, PMCdm, PMCdp, PMCvd and PMCvv) of the PMC were included as seed areas and defined separately for each hemisphere (Figure 1). The mean time series was then extracted for each PMC subregion. For each subject, the strength of the functional connectivity was measured through Pearson’s correlations between the averaged time series of each seed region and voxels in the rest of the brain. Then, the Fisher’s *z* transform was applied to normalize the original correlation maps.

After that, a two sample *t*-test (with age, gender and educational year treated as covariates) was performed on these normalized correlation maps to determine areas with significantly different connectivity to the seed region between each of the patient groups and the healthy controls. The significance was determined with a cluster-level corrected threshold of *p* < 0.05 (cluster-forming threshold at voxel-level *p* < 0.01 using the AlphaSim).

The same procedures were performed in the fMRI data without global signal regression. And the overlap parts of the brain areas with significantly different functional connectivity between the two analyses with and without global signal regression were used as the final results.

We finally used the sum of the significant areas for each subregion of the PMC and got the connection strength with that PMC subregion. To determine whether the functional connectivity changed with disease severity in the MCI and AD samples, correlation analyses were performed between the MMSE scores and the connection strength. A statistical significance level of *p* < 0.05 (FDR corrected) was used in the correlation analyses.

## Results

### Group Effect on Connectivity

**Figure 2:**
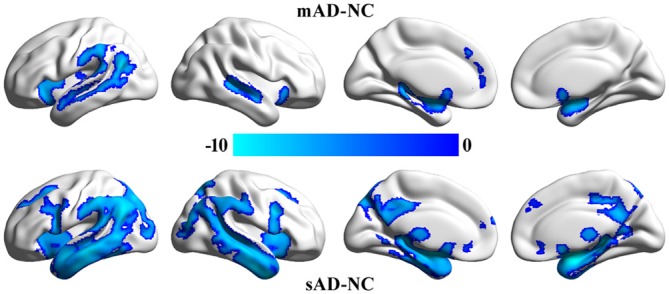
**Significant differences in gray matter volume between the patients and the healthy control group.** The whole brain voxel-wise two-sample *t*-tests were used to identify the significant differences in gray matter volume between mild cognitive impairment (MCI), mild Alzheimer’s disease (mAD), seve AD (sAD) and healthy control groups. The statistic results were corrected using family wise error (FWE) correction method (*p* < 0.05, FWE corrected).

VBM analyses identified significant differences in gray matter volume between mAD, sAD and healthy control (Figure 2). We did not find significant differences in gray matter volume between MCI and healthy control. The main differences between mAD and healthy control were found in bilateral superior temporal gyrus, superior temporal sulcus, insula, parahippocampus, and left inferior parietal lobule and medial prefrontal cortex. The main differences between sAD and healthy control were found in bilateral superior, middle, and inferior temporal gyrus, hippocampus, parahippocampus, insula, thalamus, medial prefrontal cortex, posterior cingulate cortex (PCC), precuneus, posterior inferior frontal gyrus, superior and middle frontal gyrus, and orbitofrontal cortex. The structural differences revealed an extended reducing of brain gray matter volume with the progression of AD compared with healthy control subjects.

### Group Effect on Connectivity

**Figure 3:**
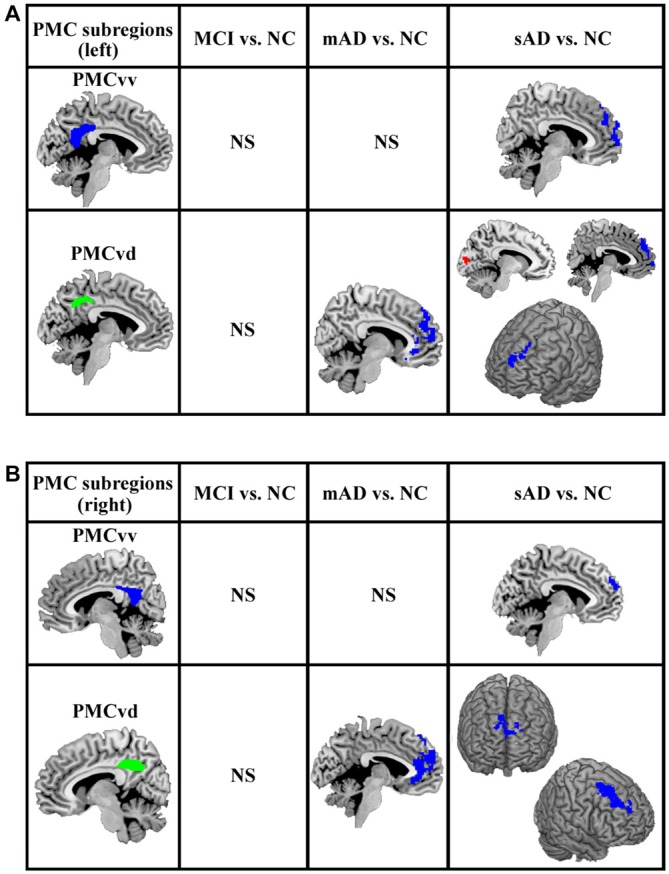
**The overlapped brain areas with significant differences in functional connectivity between the analyses with and without global signal regression.** Two-sample *t*-tests were used to identify the significant differences in functional connectivity between MCI, mAD, sAD, and healthy control groups. The significance was determined with a cluster-level corrected threshold of *p* < 0.05 (cluster-forming threshold at voxel-level *p* < 0.01 using the AlphaSim). **(A)** The changed functional connections for left PMC subregions were found under the analyses with and without global signal regression. **(B)** The changed functional connections for rihg PMC subregions were identified under the analyses with and without global signal regression. The first column shows the subregions of PMC for assessing the functional connectivity. The second, third and fourth columns show alterations in the MCI, mAD and sAD groups, respectively, compared with the healthy control group. Hot (red tones) and cold (blue) colors represent increased and decreased functional connectivity in the patient group compared with the healthy control group. NS, not significant.

The functional connectivity analyses of PMC subregions between MCI and healthy control did not find the overlapped brain areas with significantly different functional connectivity between the analyses with and without global signal regression. The overlapped brain areas with significantly different functional connections were only found between mAD, sAD and healthy control (Figure 3).

For the mAD group, decreased connectivity was observed between the bilateral PMCvd and the medial prefrontal cortex. No other overlapped brain areas were observed (Figure 3). In the sAD group, expanded subregions of the PMC with significantly altered functional connectivity were detected. Regions with changed connectivity with the bilateral PMCvd were observed primarily in frontal areas and the visual cortex, including decreased connectivity with the bilateral medial prefrontal cortex and the bilateral dorsolateral frontal cortex and increased connectivity with the cuneus. The PMCvv in left and right hemispheres both showed a pattern of reduced interaction with the medial prefrontal cortex (Figure 3).

### Functional Connectivity Strength and Cognitive Ability: Link to MMSE

**Figure 4:**
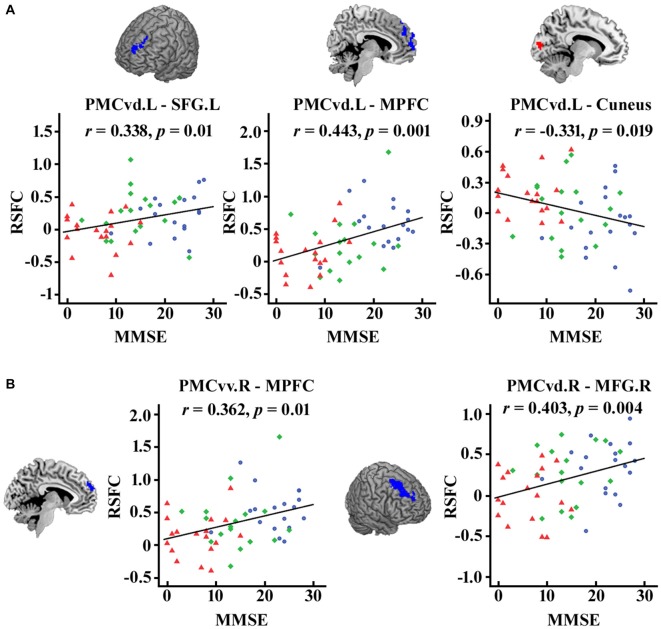
**Significant correlations between the MMSE scores and the connection strength of the PMC subregions in the patient groups.** The correlations between the MMSE scores and the changed connection strength of the PMC subregions were calculated and false discovery rate (FDR) was used for multi-comparison correction with *p* < 0.05. **(A)** Left PMC subregions **(B)** Right PMC subregions. MCI patients are indicated by blue circles, mAD patients by green rhombuses, and sAD patients by red triangles. L, left; R, right; SFG, superior frontal gyrus; MFG, middle frontal gyrus; MPFC, medial prefrontal cortex.

As illustrated in Figure 4, after FDR (*p* < 0.05) correction, we found significantly correlations between the MMSE scores and the functional connectivity of the PMC subregions, including the connection strength between the left PMCvd and the left superior frontal gyrus, between the left PMCvd and the medial prefrontal cortex, between the left PMCvd and the cuneus, between the right PMCvv and the medial prefrontal cortex, between the right PMCvd and the right middle frontal cortex.

## Discussion

In the present study, we investigated the distinct functional alterations in each of the PMC subregions throughout the progression of AD. As the severity of the AD progressed, subregions having altered functional connectivity extended from bilateral PMCvd in mAD to the bilateral PMCvv in the sAD phase. The structural analyses in our current study have also revealed that with the progression of AD, the brain areas with reduced gray matter volume extended from temporal and parietal cortex to temporal, parietal, and frontal cortex. Furthermore, the number of brain regions that showed disrupted connectivity with any given PMC subregion tended to increase with increasing disease severity.

**Figure 5:**
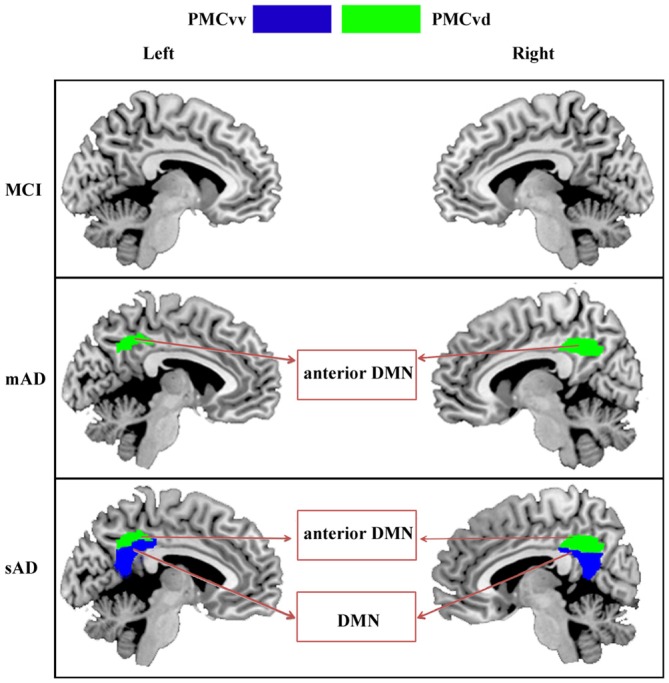
**Increasing range of changes in the PMC subregions with altered functional connectivity in the MCI, mAD, and sAD stages.** In MCI group, we did not find the overlapped brain areas with significantly different functional connectivity between the analyses with and without global signal regression. For mAD and sAD groups, in both hemispheres, the functional changes began in the PMCvd and then expanded to the surrounding subregions of the PMCvv. Distinct subregions had disrupted connectivity with different brain networks during the AD progression. Interactions between the PMC subregions and the memory, default mode, and visual networks changed gradually.

As summarized in Figure 5, the PMCvd was the main subregion of the PMC that showed dysfunction in different stages of AD. Considered to be a transition zone, the PMCvd is connected to all the other surrounding regions and also connects with several areas that are connected to the precuneus or the PCC (Cavanna, 2007; Zhang et al., 2014). Additionally, the PMCvd is thought to be the interface between the DMN and the cognitive control network (Leech et al., 2011; Xia et al., 2014). Its widespread connections and its “bridge” role may explain why the PMCvd became the most vulnerable subregion of the PMC. A functional study of MCI and AD suggested that, even when cognition was preserved, a region similar to the PMCvd exhibited deactivations that were independent of gray matter atrophy (Bosch et al., 2010). Another work demonstrated that an area in the PMCvd had lower functional connectivity, which remained significant even when using a strict correction in AD (Binnewijzend et al., 2012). Additionally, alterations in the connectivity of the PMCvd begin with decreased connectivity with the anterior medial prefrontal cortex in mAD, which was also observed in the sAD. Thus, the widespread decreased connectivity in different stages of AD can be a predictor for AD. In contrast, the connectivity between the PMCvd and the visual cortex was significantly higher in the sAD patients, a finding which was consistent with a previous functional study (Sheline et al., 2010), which further indicated that increased connectivity to the visual cortex may result in dysregulation of the DMN, providing either inhibitory input or a compensatory effect.

In the sAD group, connectivity dysfunction was also observed in the PMCvv. The PMCvv exhibited significantly reduced connectivity with the medial prefrontal cortex. As the disease state processed, connectivity between the PMCvv and the anterior nodes of the DMN was significantly lower in the AD groups. The medial prefrontal cortex is a key interface connecting the posterior and anterior DMN regions (Pomarol-Clotet et al., 2010). Its disassociation with the PMCvd may have contributed to the reduced connections between the dorsolateral frontal cortex and the PMCvd in the sAD phase. In addition, disassociation of the PMCvv from the medial prefrontal cortex may reflect widespread impairments in memory and cognition.

Previous studies have demonstrated that many clinical features of AD are associated with the PMC (Zhang et al., 2009; Pihlajamäki et al., 2010; Wu et al., 2011). The present research attempted to elucidate the potential PMC-related mechanism in AD progression. The PMC is known to be one of the earlier regions to develop amyloid plaque deposition, which is thought to be linked with the altered functional connectivity observed in the DMN (Mintun et al., 2006). Our data further suggest that the most conspicuous subregion in the PMC showing connectivity deficits was the transitional area between the precuneus and the PCC. This critical region may play a very important role in the course of AD. Additionally, the present study revealed that each of the subregions of the PMC had distinct and regular patterns in their alterations in functional connectivity throughout the progressively advanced AD states of the patients. Memory disruption in the patients was mainly associated with the PMCvd. The PMCvv was involved with alterations in the DMN. These patterns may be helpful for studying AD-related behavior changes. In addition, we assessed the appearance of the functional maps of these PMC subregions in the continuum of stages represented by the AD-affected brains. We found that the connection strength between the significant regions and PMC subregions was correlated with behavior scores. These alterations in functional networks may potentially serve as biomarkers for AD progression. They also could be used for testing the effectiveness of treatments by providing a measure of their ability to normalize functional connectivity.

There is also a limitation in our current study. Our study contains only 18 patients with MCI, 17 patients with mAD, 18 patients with sAD, and 21 healthy controls. Thus, the number of subjects is relatively small. Although all the statistic results have been corrected using multi-comparisons corrected methods, our finding needed to be further validated in a larger sample sizes in future studies.

## Conclusion

In conclusion, the present study assessed the distinct functional alterations for each of the PMC subregions with AD progression. Subregions of the PMC that showed altered connectivity started with the PMCvd, and expanded to the PMCvv. These subregions were associated with disruptions in different functional networks in AD. Connections between the PMC subregions and the memory, default mode, and visual networks seem to be affected successively.

## Author Contributions

TJ: designed and supervised the study; JL, YD, YL, KL, XZ, and CY: collected the data; YW, YZ, YL, JW, and XW: analyzed the data. YW, YZ, YL, and JW wrote the article. All authors discussed the results and commented on the manuscript. YW, YZ: contributed equally to the study.

## Conflict of Interest Statement

The authors declare that the research was conducted in the absence of any commercial or financial relationships that could be construed as a potential conflict of interest.

## Footnotes

^1^http://dbm.neuro.uni-jena.de/vbm.html

^2^http://www.fil.ion.ucl.ac.uk/spm

^3^http://afni.nimh.nih.gov/afni

^4^http://fsl.fmrib.ox.ac.uk/fsl/fslwiki/

## Abbreviations

AD: Alzheimer’s disease
CDR: Clinical Dementia Rating
DMN: default mode network
FWHM: full-width at half-maximum
MCI: mild cognitive impairment
MNI: Montreal Neurological Institute
PMC: posteromedial cortex.
